# Acute pulmonary embolism and severe post thrombolysis renal bleeding, two deadly complications following mini-percutaneous nephrolithotomy: a rare case report

**DOI:** 10.1186/s12893-022-01561-8

**Published:** 2022-03-23

**Authors:** Feng-Qi Wang, Wan-Zhang Liu, He-Sheng Yuan, Ting Huang, Zheng-Yi Wang, Jin-Feng Pan, Ze-Jun Yan, Yue Cheng

**Affiliations:** 1 Department of Urology, Ningbo First Hospital, The Affiliated Hospital of Ningbo University, #59 Liuting Street, Ningbo City, 315010 Zhejiang Province China; 2 School of Medicine, Ningbo University, No.818 Fenghua Road, Ningbo City, 315000 Zhejiang Province China

**Keywords:** Mini-percutaneous nephrolithotomy, Pulmonary embolism, Renal bleeding, D-dimer, Superselective renal artery embolization, Complication, Thrombolytic therapy

## Abstract

**Background:**

Acute pulmonary embolism and severe renal bleeding are two lethal postoperative complications, but there has been no report that involves both of them after mini-percutaneous nephrolithotomy.

**Case presentation:**

A 62-year-old woman was admitted to our hospital with extremely severe hydronephrosis and multiple right renal calculi. After thorough examination, she received prone-position mini-percutaneous nephrolithotomy under spinal anaesthesia. Three days postoperatively, the patient complained of chest pain and dyspnea. Computed tomography pulmonary angiogram (CTPA) showed multiple embolisms in the left pulmonary artery and its branches. Symptoms were relieved after anticoagulant and thrombolysis therapy. On the 6th postoperative day, the patient developed shortness of breath, computed tomography angiography (CTA) showed massive hemorrhage in the right kidney, diffused contrast medium in the middle and lower part of the right kidney was seen during digital substraction angiography (DSA). Superselective right renal artery embolization (SRAE) was then applied using coil to occlude the responsible artery. The patient generally recovered under conscientious care and was approved to be discharged 26 days postoperatively.

**Conclusions:**

This is the first case that involved both acute pulmonary embolism and severe post thrombolysis renal bleeding. The importance of D-dimer in the prediction and early detection of pulmonary embolism should be noted. For post thrombolysis renal bleeding, SRAE is considered as a reliable treatment.

## Background

Percutaneous nephrolithotomy (PCNL) is a common treatment for large upper urinary tract calculi with the advantage of both high stone-free rate and efficiency. The main postoperative complications of PCNL are bleeding, infection and organ damage [1]. Since invented, massive effort has been put to lessen PCNL-related complications, and miniaturization of the equipment is a direct and effective way. According to a recent META analysis [2], compared to standard PCNL, minimally invasive percutaneous nephrolithotomy (mini-PCNL) can reduce blood loss while maintaining a similar stone clearance rate. In the following case, the patient underwent mini-PCNL (16Fr), and then came through a consecutive process of infection, pulmonary embolism (PE), systematic thrombolysis, anticoagulation therapy, post thrombolysis bleeding and SRAE. This is the first case with two deadly complications of acute pulmonary embolism and severe post thrombolysis renal bleeding following a mini-percutaneous nephrolithotomy.

## Case presentation

**Fig. 1:**
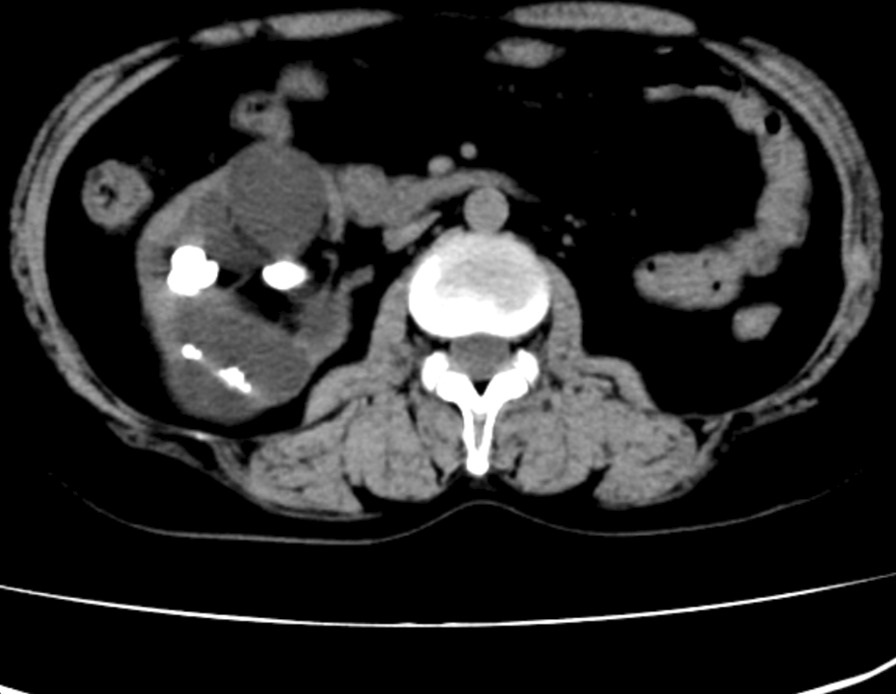
Preoperative CT scan demonstrated extremely severe hydronephrosis and multiple right renal calculi

A 62-year-old woman came to our center with extremely severe hydronephrosis and multiple right renal calculi found by annual physical check. She reported no renal colic, nausea or fever and had no current use of any medications. Her temperature was 36.7 °C, pulse 89 bpm, respiratory rate 18 bpm, and non-invasive blood pressure 130/57 mmHg. Unenhanced CT scan demonstrated extremely severe hydronephrosis and multiple right renal calculi with the largest diameter of 2 cm (Fig. 1). Urine routine was positive for nitrites and leukocyte esterase, and microscopical examination showed + + + for leucocyte.

After thorough examination, we performed prone-position mini-percutaneous nephrolithotomy under spinal anaesthesia. A single tract was created under the guidance of ultrasound to directly reach the median calyceal group. After the insertion of a guidewire, the channel was dilated to 16Fr with a fascia dilator. During the operation, swollen mucosa could hardly be seen through turbid purulent urine. Multiple golden stones embedded in the renal pelvis were fragmented with holmium laser and flushed out, and a considerable number of the stones were removed. After 2 h’ lithotripsy, the operation finished after successful indwelling of a 6Fr double-J stent and a 16 F nephrostomy tube.

Two hours postoperatively, the patient complained of chilly and shiver. Her blood pressure dropped to 85/60 mmHg, and blood tests showed C-reactive protein 31.4 mg/L, White blood cell 9.55 × 10^9^/L, D-dimer 570.0 ng/ml. Symptoms were relieved after empirically intravenous infusion of 300 mg biapenem bid and micro-pump injection with dopamine.

**Fig. 2:**
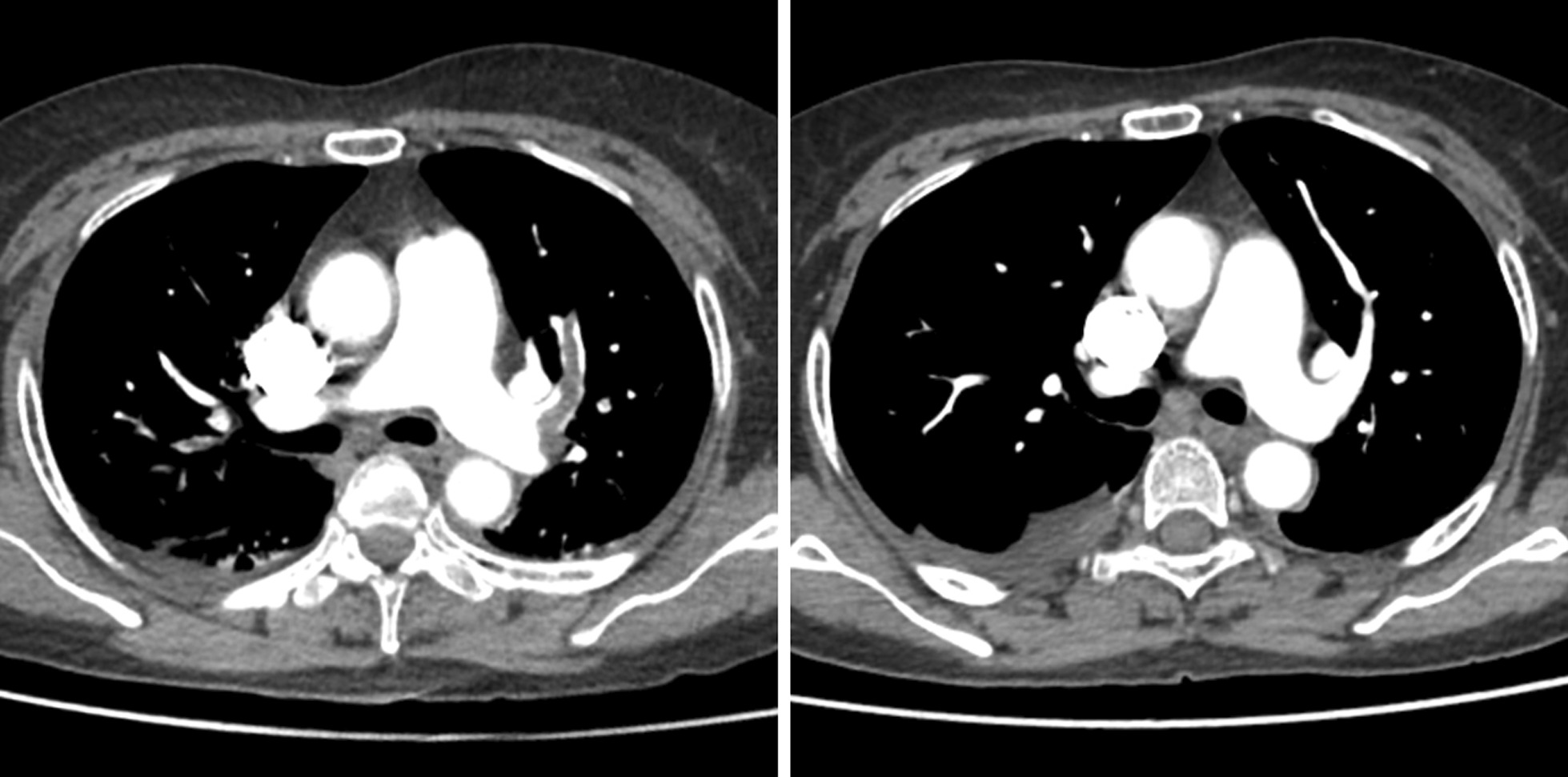
**a** Emergency CTPA showed an embolism occluding the patient’s left pulmonary artery. **b** 1 month later, CTPA showed the patient’s left pulmonary artery has recanalized

Three days later during the CT scan, the patient abruptly developed respiratory distress and loss of consciousness. Her blood pressure dropped to 76/45 mmHg, and oxygen saturation decreased to 85% under nasal catheter inhalation of oxygen. Clinical findings indicated the possibility of PE with shock. Supported by multi-channel intravenous rehydration, micro-pump injection with norepinephrine and mask oxygen inhalation, her blood pressure fluctuated between 108–124 and 65–78 mmHg. However, the patient still complained of dyspnea and chest pain, and her blood oxygen saturation remained unstable at an average of 92% under mask oxygen inhalation. Emergency laboratory tests showed a D-dimer of more than 3300 ng/ml. Considering the rising level of D-dimer and the patient’s clinical manifestation, we decided that PE might be the most likely diagnosis. Thus, we administrated anticoagulant therapy with 4000 U low molecular weight heparin (LMWH) at once and offered emergency CTPA, which revealed multiple embolisms in her left pulmonary artery and its branches (Fig. 2). Meanwhile, doppler echocardiography showed that the patient’s left ventricular ejection fraction (LVEF) was 52% with severe tricuspid regurgitation, and experienced sonographers estimated that pulmonary arterial systolic pressure was about 40 mmHg (mild pulmonary hypertension). Doppler ultrasonography of lower limbs revealed intermuscular venous thrombosis in her left leg.

It was a contradictory situation with both the urgent need for thrombolysis therapy and the high vulnerability of surgery field bleeding. In order to solve the life-threatening problem first, our multi-disciplinary team administrated 50 mg of Alteplase ivgtt which was completed within 2 h for thrombolysis, and then transferred the patient to intensive care unit (ICU) for further life-supporting therapy. In order to prevent venous thromboembolism (VTE), 0.4 ml LMWH was subcutaneously injected every 12 h in ICU. Routine re-examination of the lower limbs venous ultrasonography and echocardiography was ordered to monitor the patient’s condition. Sonographers reported the remission trend of tricuspid regurgitation and pulmonary hypertension. Intermuscular venous thrombosis in her left leg could no longer be detected 6 days after the thrombolysis treatment.

**Fig. 3:**
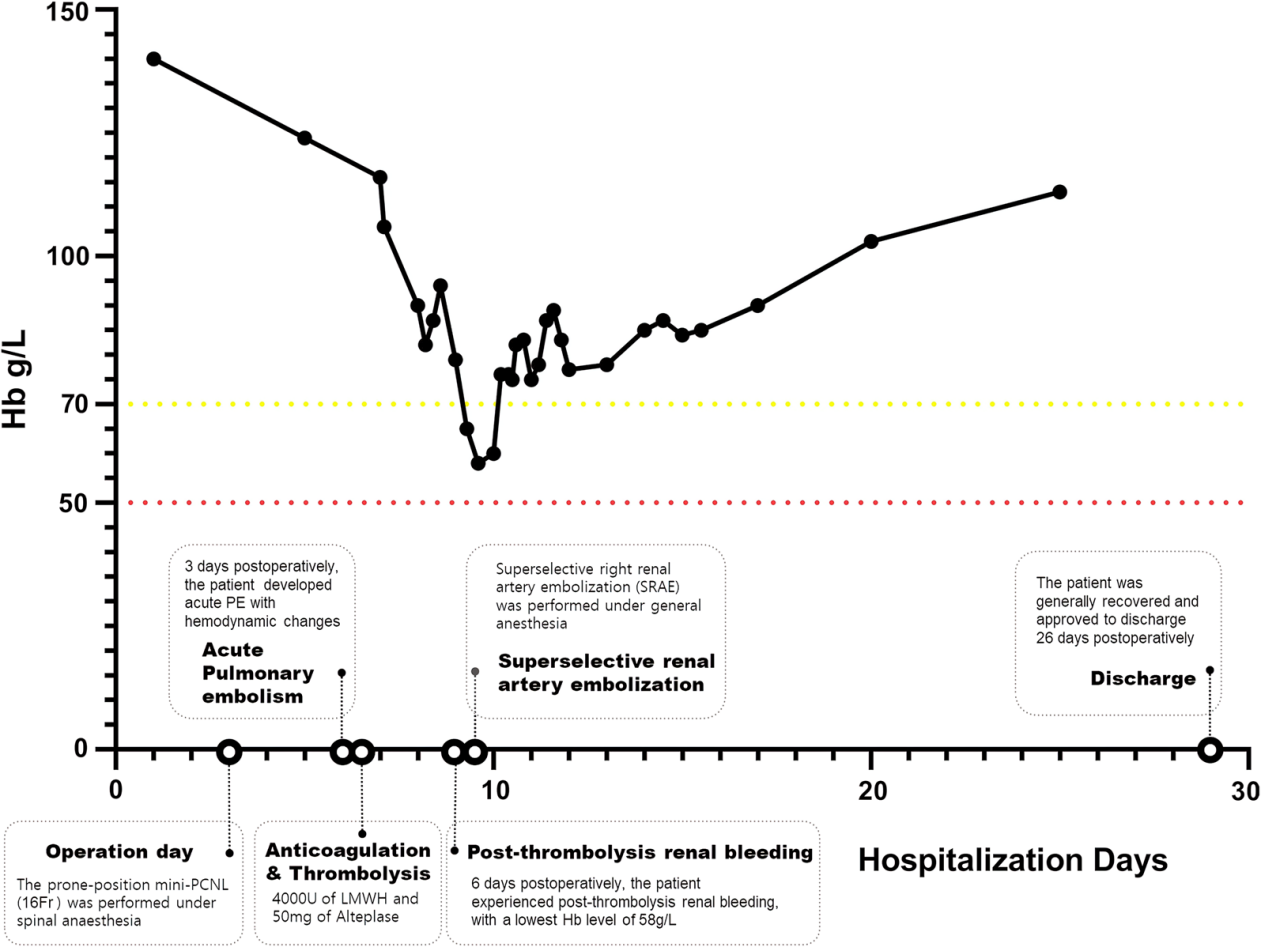
The line graph showing the treatment stages and fluctuation of patient’s haemoglobin (Hb) level

**Fig. 4:**
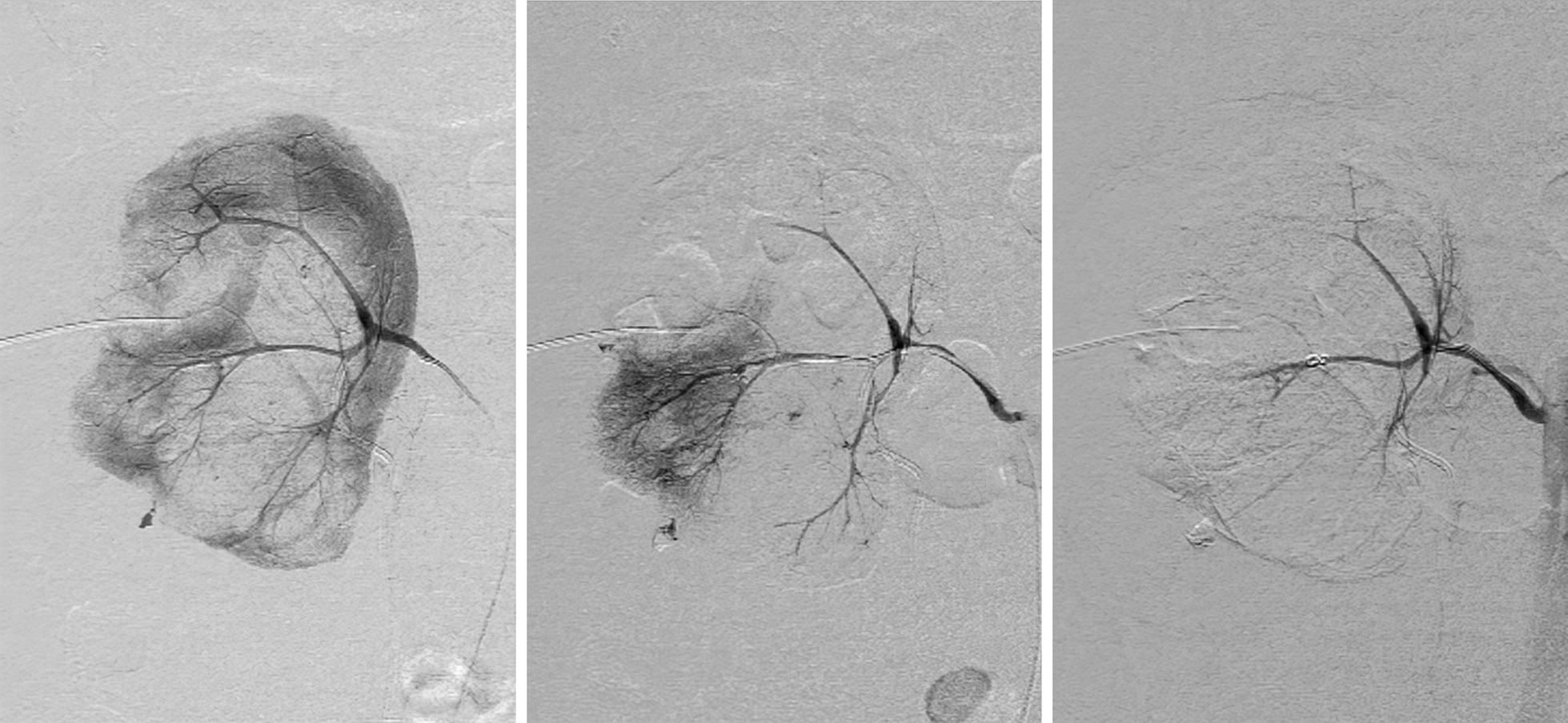
**a, b** The DSA demonstrated the injured vessels and diffused contrast medium in the middle and lower part of the right kidney. **c** The post-SRAE image showed the responsible arteries were occluded

On the 6th postoperative day, the patient suddenly developed unconsciousness and anhelation in ICU, and her blood oxygen saturation progressively dropped to 85%. Urgent intubation, mechanical ventilation and blood transfusion were applied to correct the patient’s anemia. Gradually, her oxygen saturation rose to 98%, but crimson and opaque drainage fluid kept flowing through her nephrostomy tube without any sign to stop even after clamping the catheter for 30 min. Her hemoglobin (Hb) level was still decreasing progressively (Fig. 3). All the above clues were indicative of post thrombolysis bleeding. Diffused contrast medium in the middle and lower part of the right kidney was seen during DSA (Fig. 4). SRAE was then performed under general anesthesia. The patient reported no obvious discomfort and generally recovered after conventional symptomatic treatment and blood transfusion. Anticoagulant treatment method was gradually changed to oral application of Warfarin 3 mg qn, and the patient was approved to be discharged 26 days postoperatively. She returned to her daily activity and reported no obvious discomfort, and during re-examination, the echocardiography showed LVEF of 70% with no obvious abnormality.

## Discussion and conclusion

Pulmonary embolism (PE) is a type of VTE. It is a relatively rare but life-threatening complication for patients undergoing PCNL: The dense distribution of blood vessels in the operating field, intraoperative vascular wall injury, and the increase in bed rest time might all contribute to the rising incidence of post-PCNL PE [3]. The previous article reported PE after conventional PCNL (30Fr) and discharged more than 2 months postoperatively [4]. In this case, the patient underwent mini-PCNL (16Fr), followed by a consecutive process of infection, PE, anticoagulant and systemic thrombolytic therapy and post thrombolysis bleeding. This is the first case in our center since 2015 among more than 2000 mini-PCNL (16Fr) procedures.

In terms of thromboprophylaxis, EAU2020 guidelines [5] recommended mechanical prophylaxis and pharmacological prophylaxis to prevent VTE. When it comes to PCNL, EAU Guidelines recommended using neither mechanical nor pharmacological way to prevent thrombosis. Mechanical prophylaxis was offered only for high-risk VTE patients (weak). In clinical practice, the combination of mechanical and pharmacological prevention for VTE was adopted in our center. VTE prevention education was routinely carried out for all patients, and early out-of-bed activities were also advocated. Patients with intermediate and high risk wore gradient compression socks or received air pressure therapy postoperatively.

In this case, the elevation of D-dimer (570 ng/ml) 2 days after the surgery drew our attention, so we informed the attendants to massage the lower limbs of the patient to prevent thrombosis, and dynamically re-examined the D-dimer. In our daily practice, D-dimer was re-examined the first day after surgery, and patients with elevated D-dimer (> 500) were offered with ultrasound of the lower extremity vein. A previous study [6] demonstrated that D-dimer combined with age threshold could be used in low and intermediate-risk PE patients to minimize unnecessary tests: for patients older than 50, their D-dimer positive threshold could be adjusted to their age multiplied by ten. Therefore, to detect PE as soon as possible, urologists should pay close attention to the results of D-dimer for its high sensitivity to VTE and PE. For suspicious PE patients, every minute counts, so early diagnosis may provide the time-window for more intervention choices that may improve the prognosis.

Although D-dimer has an ideal sensitivity in the diagnosis of PE, its specificity varies. Renal function abnormality is common in patients with urinary calculi, but a previous study has shown that the specificity of D-dimer to rule out PE decreases with declining renal function [7]. Thus, when positive serum D-dimer result triggers the alert, immediate CTPA is needed to establish the PE diagnosis. CTPA provides excellent accuracy in PE diagnosis and also contributes to the differential diagnosis with other cardiovascular or pulmonary diseases. The risk of PE could also be estimated through right ventricle (RV) dilation with other approaches like echocardiography, although it may not deliver the direct diagnosis. Once confirmed, oxygen inhalation, anticoagulation and other treatments should be implemented as soon as possible in order to obtain higher safety and shorter recovery time.

According to the 2019 edition of ESC-APE guidelines, anticoagulant therapy is the primary treatment for acute pulmonary embolism, while thrombolysis therapy may ameliorate pulmonary artery pressure (PAP), pulmonary vascular resistance (PVR) and RV dilatation by promoting the dissolution of blood clots in high-risk patients with hemodynamic changes [8]. A meta-analysis concludes that compared with single anticoagulation therapy, the combination of anticoagulation and thrombolysis therapy could reduce all-cause mortality, lower the PE recurrence rate and PE-related mortality in hemodynamic instable PE patients (high risk), but the risk of massive hemorrhage increases by 10% [9]. Intermediate-risk patients with right ventricular dysfunction could also benefit from thrombolysis therapy for lowering all-cause mortality with the combination of anticoagulation and thrombolysis therapy [10]. According to previous studies, the ideal thrombolysis time-window for PE is 48 h after the onset of symptoms, but patients can still benefit from thrombolysis treatment within 6–14 days. Earlier thrombolysis therapy might result in a higher recanalization rate. Therefore, applying thrombolysis therapy as early as possible for medium and high-risk PE patients may improve their prognosis [11].

For those patients who completed percutaneous nephrolithotomy, surgical field bleeding after thrombolysis is considered nearly inevitable. We believed that early thrombolysis and accurate hemostasis of the relevant vessels might shorten the recovery time, reduce blood loss and lower the risk of hemorrhagic shock. The color of urine and Hb level are vital signs that might contribute to the early diagnosis of post thrombolysis bleeding. To the best of our knowledge, progressive bleeding can be preliminarily judged by the drainage fluid and the level of Hb: The appearance of the drainage fluid in the nephrostomy tube is crimson and opaque even after clamping the tube for a while; The level of Hb keep decreasing progressively. For patients with those signs, DSA could help to make the diagnosis and directly block the injured vessels by SRAE. It could precisely locate and reliably embolize the injured vessels to control bleeding while minimizing the influence of systemic coagulation function. For those who failed to stop bleeding after one SRAE treatment, repeated SRAE is still an effective backup plan. The result of a retrospective study involving 49 patients who underwent SRAE found no significant decrease in renal function during the 6–64month follow-up compared with preoperative examinations [12]. For patients with post thrombolysis bleeding, we believe that SRAE is still effective, even for dispersed bleeding. Take this case as an example, DSA showed diffused contrast medium in the middle and lower part of the right kidney. After SRAE with coil, the responsible artery was occluded. The patient reported no obvious discomfort after SRAE, and Hb stopped decreasing. Through anticoagulant therapy, blood transfusion and other symptomatic treatment, her Hb level returned to the normal level, and the patient was approved to be discharged 26 days postoperatively.

To sum up, for patients who develop PE after percutaneous nephrolithotomy, early diagnosis, prompt anticoagulation therapy and appropriate thrombolysis are necessary. Since post-thrombolysis bleeding is nearly inevitable for patients who underwent PCNL, close monitoring of post-thrombolysis bleeding is vital to minimize blood loss and prevent hemorrhagic shock. To the best of our knowledge, immediate SRAE might be the ideal treatment for its adequate hemostatic ability with scanty impact on renal function. More importantly, urologists should be cautious in surgical decision-making, especially for patients with a solitary kidney or renal insufficiency. Although mini-PCNL could achieve less bleeding compared with conventional PCNL, strategic staging retrograde intrarenal surgery might be an alternative for better safety.

## Data Availability

Not applicable.

## Abbreviations

CTA: Computed tomography angiography
CTPA: Computed tomography pulmonary angiogram
DSA: Digital subtraction angiography
Fr: French
Hb: Hemoglobin
ICU: Intensive care unit
LVEF: Left ventricular ejection fraction
LMWH: Low molecular weight heparin
Mini-PCNL: Minimally invasive percutaneous nephrolithotomy
PCNL: Percutaneous nephrolithotomy
PE: Pulmonary embolism
RV: Right ventricle
SRAE: Superselective renal artery embolization
VTE: Venous thromboembolism
